# Production of Melanins With Recombinant Microorganisms

**DOI:** 10.3389/fbioe.2019.00285

**Published:** 2019-10-24

**Authors:** Luz María Martínez, Alfredo Martinez, Guillermo Gosset

**Affiliations:** Departamento de Ingeniería Celular y Biocatálisis, Instituto de Biotecnología, Universidad Nacional Autónoma de México, Cuernavaca, Mexico

**Keywords:** melanin, metabolic engineering, aromatics, tyrosinase, process engineering

## Abstract

The melanins constitute a diverse group of natural products found in most organisms, having functions related to protection against chemical and physical stresses. These products originate from the enzyme-catalyzed oxidation of phenolic and indolic substrates that polymerize to yield melanins, which include eumelanin, pheomelanin, pyomelanin, and the allomelanins. The enzymes involved in melanin formation belong mainly to the tyrosinase and laccase protein families. The melanins are polymeric materials having applications in the pharmaceutical, cosmetic, optical, and electronic industries. The biotechnological production of these polymers is an attractive alternative to obtaining them by extraction from plant or animal material, where they are present at low concentrations. Several species of microorganisms have been identified as having a natural melanogenic capacity. The development and optimization of culture conditions with these organisms has resulted in processes for generating melanins. These processes are based on the conversion of melanin precursors present in the culture medium to the corresponding polymers. With the application of genetic engineering techniques, it has become possible to overexpress genes encoding enzymes involved in melanin formation, mostly tyrosinases, leading to an improvement in the productivity of melanogenic organisms, as well as allowing the generation of novel recombinant microbial strains that can produce diverse types of melanins. Furthermore, the metabolic engineering of microbial hosts by modifying pathways related to the supply of melanogenic precursors has resulted in strains with the capacity of performing the total synthesis of melanins from simple carbon sources in the scale of grams. In this review, the latest advances toward the generation of recombinant melanin production strains and production processes are summarized and discussed.

## Introduction

The melanins comprise a group of polymeric pigments that are widely found in nature (d'Ischia et al., 2015). These are the result of the enzyme-catalyzed oxidation of phenolic or indolic substrates. The melanins are considered one of the most ancient pigments found in nature. These pigments have been detected in fossils of birds and dinosaurs (Zhang et al., 2010). Remarkably, preserved melanin was found in cephalopod ink sacs from the Jurassic period (Glass et al., 2012). Thus, melanin is proposed as a biomarker to study evolution (Wogelius et al., 2011).

**Figure 1:**
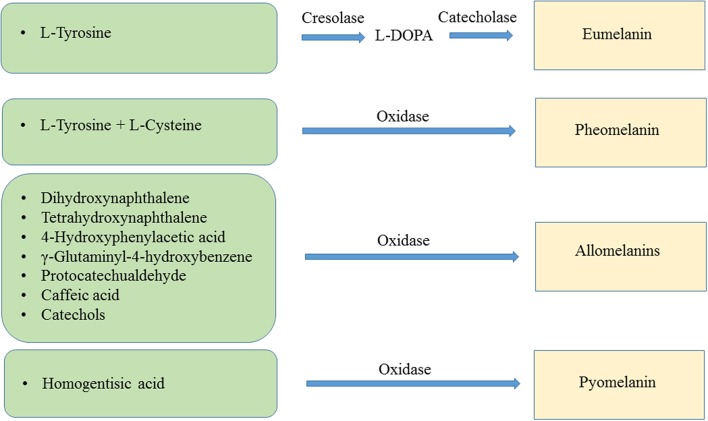
Biochemical reactions leading to the synthesis of eumelanin, pheomelanin, allomelanins, and pyomelanin.

The main types of melanin are eumelanin, pheomelanin, the allomelanins and pyomelanin. Eumelanin is the product of the oxidation of the amino acid L-tyrosine and/or L-dihydroxyphenylalanine (L-DOPA). The resulting polymer displays a brown or black color. Pheomelanin is produced when L-tyrosine and/or L-DOPA are oxidized in the presence of L-cysteine, resulting in pigment with a red-yellow color. The allomelanins are the result of oxidation of either one of the following compounds: 4-hydroxyphenylacetic acid, catechols, dihydroxynaphthalene (DHN), γ-glutaminyl-4-hydroxybenzene or tetrahydroxynaphthalene, protocatechualdehyde, and caffeic acid. Pyomelanin is a type of melanin resulting from the oxidation of homogentisic acid (HGA) (Figure 1) (Lindgren et al., 2015).

In humans and many mammals, eumelanin and pheomelanin are the prevalent skin pigment. Skin pigmentation has been a subject of interest since ancient times. There are references to diseases affecting skin color, such as vitiligo, dating back to the year 2200 BC. It was until the year 1819 that pigment cells, called chromatophores, were described in studies with the squid. A few years later, similar structures were recognized in human skin and eyes. The term melanin was used for the first time by C. P. Robin in 1873 and later, the specialized cells responsible for melanin synthesis in the skin, the melanocytes, were identified. Further studies in the following years established the existence of melanin grains in the melanocytes and the process for the transfer of these structures to the epithelial cells (Westerhof, 2006).

As a result of their chemical composition, the melanins display distinct physicochemical properties. Thus, these polymers can act as ultraviolet light, X-ray and γ-ray absorbers, cation exchangers and amorphous semiconductors (Sarna et al., 1976; della-Cioppa et al., 1990; Krol and Liebler, 1998; Rózanowska et al., 1999; Ambrico et al., 2014). Melanins have also been shown to have antioxidant and antiviral activities (Montefiori and Zhou, 1991; Nofsinger et al., 2002). Diverse applications and products derived from melanins are dependent on obtaining these polymers at a relatively low cost and in a large quantity. Melanins can be extracted from plant and animal tissues, or generated by chemical synthesis. However, these processes are relatively expensive and in some cases, not sustainable (Saini and Melo, 2015). A potentially viable alternative to obtain melanins is based on the culture of melanogenic microorganisms. This method has the advantage of being scalable and providing a good yield of melanins. This approach can be improved by applying genetic engineering techniques to increase the natural melanogenic capacity of some organisms or generating novel melanin-producing strains. The most common genetic modification to enhance/generate a production strain involves the expression of genes encoding the enzymes involved in the oxidation of melanin precursors.

## Enzymes Involved in Melanin Formation

The enzyme-dependent oxidation of phenolic or indolic compounds is the first step leading to the generation of the melanins. Melanogenic enzymes belong mainly to the tyrosinase and laccase protein families. The tyrosinases are the most common type of enzyme associated with melanogenesis. These enzymes can employ both mono and diphenolic compounds as substrates. Examples of these substrates are L-tyrosine, L-DOPA, and catechols. The tyrosinases are mono-oxygenases having a dinuclear copper catalytic center. These enzymes catalyze the ortho-hydroxylation of monophenols (cresolase activity) and also the oxidation of catechols (catecholase activity), generating ortho-quinone products (Garcia-Molina et al., 2007) (Figure 1). The enzyme tyrosinase catalyzes the hydroxylation of L-tyrosine to L-DOPA using molecular oxygen and then oxidizes this compound to dopachrome, which non-enzymatically polymerizes to yield melanin (Ito, 2003). Based on their amino acid sequence and functional features, microbial tyrosinases can be divided into five main groups (Fairhead and Thöny-Meyer, 2012). The tyrosinase from *Streptomyces* sp. is included in one of these groups. They have in common the requirement of a chaperone protein that inserts copper atoms into the active site of the tyrosinase. In contrast, the tyrosinases from bacteria, such as *Rhizobium etli, Bacillus megaterium*, and *Bacillus thuringiensis*, do not require a chaperone for copper insertion into the active site. The lacasses are another group of enzymes involved in melanogenesis. These enzymes are not related to the tyrosinases but are also copper-dependent oxidoreductases. The laccases have been found in bacteria, fungi, and plants (Valderrama et al., 2003). The enzyme 4-hydroxyphenylacetic acid (4-HPA) hydroxylase is involved in the catabolism of 4-HPA in bacteria. This group of enzymes displays a broad substrate range, they can hydroxylate various monohydric and dihydric phenols (Prieto et al., 1993). 4-HPA hydroxylase is a two-component flavin adenine dinucleotide (FAD)-dependent monooxygenase (Gibello et al., 1995).

## Biological Functions of Melanins

**Figure 2:**
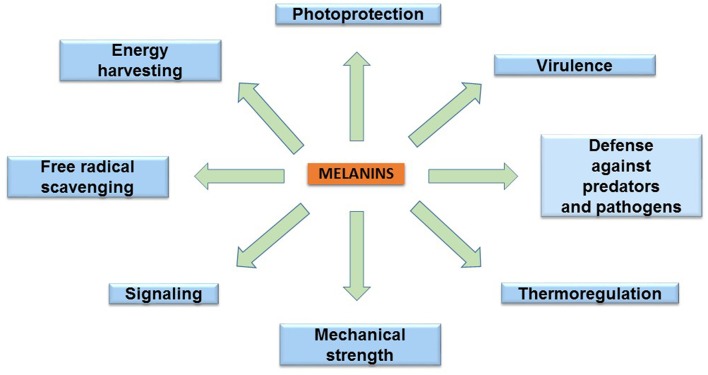
The main biological functions of melanins.

The melanins are found in species of the three domains of life: Archaea, Bacteria, and Eukarya. These pigments have diverse functions related to the survival of many species in their natural environment (Figure 2). In humans, eumelanin and pheomelanin are involved in protection against UV radiation (Coelho et al., 2009). Another important protective activity of these pigments includes their functions as free radical scavengers. This activity reduces the production of reactive oxygen species (Meredith and Sarna, 2006). Melanin is also found in the eyes and brain of humans and other vertebrates. However, the role of the pigment in these organs is not completely understood. In birds, melanin is involved in feather coloring. This function is related to signaling, having an impact on reproductive fitness (McGraw, 2008). The dark color imparted by melanin serves a function in thermoregulation by absorbing radiant energy in organisms, such as amphibians and reptiles (Clusella-Trullas et al., 2007). In some species of the molluscs octopus and squid, the production and secretion of ink is a distinctive defense mechanism. The main constituent of this product is eumelanin, which is synthesized by an ink gland in these organisms (Palumbo, 2003). In insects, melanin formation is related to cuticle sclerotization. The cuticle is the outer component of the exoskeleton of insects. Melanogenesis leads to the hardening of the cuticle, providing protection against physical damage. In addition, melanization functions as a defense mechanism against pathogens in insects. Upon infection, melanin formation around a pathogen blocks its proliferation (Vavricka et al., 2014). In fungi, melanization is a common trait that is related to pathogenesis. In these organisms, melanin precursors include DHN, HGA, γ-glutaminyl-4-hydroxybenzene, catechol and tyrosine. In addition to photoprotection and antioxidant activities, in fungi, melanins are also involved in providing resistance against chemical and mechanical stresses (Cordero and Casadevall, 2017). Furthermore, melanin has been proposed as an energy harvesting pigment in fungi. In has been determined that sub-lethal doses of gamma rays cause an enhanced increase in NADPH levels and rate of growth in several fungi species (Dadachova et al., 2007). Melanin production by bacteria has been identified in species from *Rhizobium, Streptomyces, Marinomonas, Pseudomonas, Serratia*, and *Bacillus*. In these organisms, melanin is involved in virulence, as well as protection against ultraviolet light and oxidation agents (Trias et al., 1989; Patel et al., 1996; López-Serrano et al., 2004; Piñero et al., 2007; Manivasagan et al., 2013).

## Applications of Melanins

The melanins have a very complex polymeric structure, resulting in diverse chemical and physical properties. In addition to blocking UV light, they can also absorb X and γ-rays (Hill, 1992). These polymers also have the capacity of scavenging reactive oxygen species and free radicals, as well as exhibiting redox behavior (Liu et al., 2015). Melanin is an amorphous semiconductor, as such, it is being evaluated as a component of electronic circuits, batteries as well as solar cells (Bothma et al., 2008; Kim et al., 2013; Ambrico et al., 2014). Inorganic semiconductors are currently being employed for these applications. However, they have a high environmental impact and relatively high cost. In contrast, organic semiconductors, such as melanins, do not have the same drawbacks and are easier to process. An additional advantage of melanin over traditional semiconductors is its biocompatibility, making it suitable to be used in implantable devices.

In another type of application, melanin has been employed as a template to synthesize silver or gold nanostructures and nanoparticles, having potential uses in the food and health industries (Apte et al., 2013; Patil et al., 2018). Melanin has also been evaluated as an additive of a synthetic polymer. The addition of eumelanin to poly(methyl methacrylate) (PMMA) was observed to cause a significant increase in thermal stabilization (Shanmuganathan et al., 2011). Further studies will be required to show if melanins can be employed to enhance the properties of other synthetic polymers. In a related study, it was demonstrated that allomelanin could be incorporated as a dye to hydrogel of soft contact lenses (Ahn et al., 2019). As compared to synthetic dyes, the use of allomelanin offers the advantage of antibacterial and antioxidant activity. In the medical field, it has been reported that *Escherichia coli* cells expressing the *melA* gene encoding tyrosinase from *Rhizobium etli* can be employed for photoacoustic imaging, a method with improved depth-to-resolution ratio when compared to optical imaging (Paproski et al., 2015). This approach holds the potential of improving the understanding of bacterial pathogenic processes. In another imaging application, melanin has been employed as a contrast agent in magnetic resonance imaging probes (Williams, 1994). Dermal and cosmetic applications of melanin include its use for hair dyeing. The widely used synthetic oxidative dyes cause hair damage and are not easy to handle. In contrast, a process based on the use of melanin precursors that can bind to hair after air oxidation has the advantage of not causing damage and being safer (Koike and Ebato, 2013).

The melanins can act as metal chelators and this capacity can be employed in environmental applications. The binding of metals to melanin involves multiple coordination bonds between the hydroxyl, amine and carboxyl functional groups in this polymer. In a soil bioremediation study, melanin from fungi has been shown to efficiently bind heavy metals, such as zinc and lead (Fogarty and Tobin, 1996). In another study, melanin was synthesized by employing a tyrosinase extracted from the plant *Amorphophallus campanulatus* and L-DOPA as substrate. It was determined that melanin could efficiently remove uranium from an aqueous solution (Saini and Melo, 2013).

It should be noted that the previous cases are still in the development stage and have not yet been commercialized. However, there are a few examples of melanin-containing products that are commercially available. At present, the main commercial application of melanin is as a dye in lenses of sunglasses. In this case, it is not known which are the chemical dyes replaced by melanin, but the natural origin of the pigment and the capacity to reduce high energy visible light are highlighted as an advantage (https://espeyewear.com/). A commercial product related to dermatology is a sunscreen for dry skin containing squid ink as an antioxidant. An advantage of this product over competing sunscreens is the expected reduced irritation on the skin when compared to synthetic dyes (https://chicet.com/product/melanin-sunscreen-for-dry-skin/).

## Production of Melanins With Natural Melanogenic Organisms

The current and potential applications with melanins are dependent on the possibility of obtaining these pigments from abundant and relatively inexpensive sources. These products can be extracted from natural sources, such as animal or plant tissues by following relatively inexpensive methods. However, these sources usually contain a mixture of different types of melanins and related substances, which complicate purification procedures and might yield a product of variable composition. These polymers can also be obtained by either the chemical or enzymatic oxidation of phenolic or indolic substrates (Saini and Melo, 2015). These methodologies can generate melanins with a high degree of purity but at a relatively high cost. Another option for obtaining these polymers is based on the culture of natural melanin-producing microbes or microbes that have been genetically engineered to produce melanins. This approach has the potential for generating this class of products with a relatively low cost and high yield.

Although this review focuses mainly on engineered microorganisms, a brief description of efforts toward the development of melanin production processes with natural melanogenic organisms is included. The production of melanin has been observed in several species of microorganisms and fungi both in their natural environment an under laboratory growth conditions. Species of organisms with melanogenic capacity that have been employed for developing production processes include *Pseudomonas stutzeri, Gliocephalotrichum simplex, Rhizobium* sp., *Brevundimonas* sp., *Aspergillus fumigatus, Bacillus safensis, Streptomyces lusitanus*, and *Streptomyces kathirae* (Jalmi et al., 2012; Zhao and Tong-Suo, 2012; Ganesh Kumar et al., 2013; Surwase et al., 2013; Guo et al., 2014; Madhusudhan et al., 2014; Tarangini and Mishra, 2014; Raman et al., 2015). Processes for obtaining melanin with these organisms usually involves statistical experimental methods aimed at identifying culture conditions and media components that positively impact the productivity (Zhao and Tong-Suo, 2012; Tarangini and Mishra, 2014). Culture parameters, such as temperature, pH, oxygen, and melanin precursors concentrations have been found to contribute to productivity. The developed processes have enabled the production of melanin at titers that span 0.01–13.7 g/L (Guo et al., 2014; Raman et al., 2015). In most of these processes, a positive correlation with polymer production was observed by increasing in culture media the amount of L-tyrosine or components that contain it. Thus, the polymer produced is likely eumelanin. However, in most cases, culture media includes yeast extract or protein hydrolysates. Therefore, during the melanin formation process, some media components in addition to L-tyrosine can be incorporated into the polymer, yielding a pigment that is not pure eumelanin. This is an important drawback of most processes developed with melanogenic organisms that require complex media for growth and production.

## Production of Melanins With Genetically Engineered Microorganisms

The experimental methodologies collectively known as genetic engineering techniques allow the modification of the genetic material of microbes with the purpose of enhancing or generating the capacity to produce specific molecules. It is possible currently to genetically engineering diverse microorganisms and this number is continuously growing. The application of DNA sequencing technology combined with biochemical analyses has permitted the elucidation of pathways and specific genes related to the production of melanins. This knowledge and technologies are the basis for generating recombinant microbes for enhanced melanin production and for transferring this capacity to non-melanogenic microorganisms.

## Generation of Melanogenic Microorganisms by Expression of Genes Encoding Tyrosinases

What follows is a review and analysis of advances related to the generation of recombinant microbial strains and production processes for the synthesis of melanins. The first example of a recombinant melanogenic microbe was reported with the bacterium *E. coli*. This organism was modified to express genes from the actinomycete *Streptomyces antibioticus*. In *S. antibioticus*, the *mel* locus includes two genes, *mel*, and ORF438, that are required for melanin production. The recombinant *E. coli* strain was shown to produce eumelanin from L-tyrosine in agar plates and liquid cultures but the titers were not reported. Interestingly, it was also demonstrated that synthetic non-natural amino acids, such as N-acetyl-L-tyrosine and L-tyrosine ethyl ester could be taken as substrates by the *S. antibioticus* tyrosinase, yielding synthetic melanins (della-Cioppa et al., 1990). In another report, the *mel* locus from *S. antibioticus* was also employed to generate a recombinant *E. coli* strain derived from JM109. The gene *mel* was placed under transcriptional control of the phage T5 promoter and two *lac* operators. Culturing this recombinant strain in LB medium resulted in the recovery of 0.4 g/L of eumelanin (Table 1). The recovery of eumelanin from the culture medium was based on precipitation by adjusting pH to 3.0, followed by dissolving it in distilled water at pH 8.0. This procedure was followed by liquid chromatography on Sephadex LH-20. The purified eumelanin was employed to study the effect of the presence of this polymer on the antimicrobial activity of several antibiotics. It was determined that eumelanin reduced the antibiotic effect on *E. coli* of ampicillin, kanamycin, polymyxin B, and tetracycline in a dose-dependent manner (Lin et al., 2005). In addition to the clinical importance of such results, the observed response could be employed to select higher melanin-producing recombinant strains, based on antibiotic resistance.

**Table 1:** Engineered microbial strains for the production of melanins.

**Promoter**	**Inducer**	**Expressed gene(s)**	**Expression vectors**	**Genes origin**	**Production organism**	**Melanin precursor**	**Carbon source**	**Process temperature**	**Volumetric productivity (mg/L/h)**	**Titer (g/L)**	**References**
*lac*	Not reported	*mel*	pGEM-7Zf	*Bacillus thuringiensis 4D11*	*Escherichia coli*	Casein	Casein	Not reported	155.5	5.6	Ruan et al., 2005
T5	IPTG 0.36 mM	*mel*	pQE32	*Streptomyces antibioticus*	*Escherichia coli*	L-tyrosine	LB medium	37°C	8.3	0.4	Lin et al., 2005
*trc*	IPTG 0.1 mM	*MutmelA*	pTrc99A	*Rhizobium etli*	*Escherichia coli*	L-tyrosine	Glucose	30°C	75	6	Lagunas-Muñoz et al., 2006
None	None	None	None	*Pseudomonas putida* strain F6	*Pseudomonas putida* strain F6-HDO	L-tyrosine	Citrate	30°C	17.5	0.35	Nikodinovic-Runic et al., 2009
P_skmel_	Constitutive	*melC*	pIJ86	*Streptomyces kathirae*	*Streptomyces kathirae*	L-tyrosine	Amylodextrine, yeast extract	28°C	225	28.8	Guo et al., 2015
None	None	Not identified	None	*Escherichia coli*	*Escherichia coli*	Caffeic acid	Glucose	30°C	16.7	0.15	Jang et al., 2018
T7	IPTG 1 mM	*fcs*	pRSF duet-1 pET duet-1	*Burkholderia glumae* BGR1	*Escherichia coli*	Caffeic acid	Glucose	30°C		0.20	Jang et al., 2018
T7	IPTG 1 mM	*ech*		*Burkholderia glumae* BGR1							Jang et al., 2018
*lac*	IPTG 0.1 mM	*aroG^*fbr*^*	pTrc99A	*Escherichia coli*	*Escherichia coli*	None	Glucose	30°C	26.8	3.2	Chávez-Béjar et al., 2013
*trc*	IPTG 0.1 mM	*tyrC*		*Zymomonas mobilis*							Chávez-Béjar et al., 2013
*trc*	IPTG 0.1 mM	*pheA_*CM*_*		*Escherichia coli*							Chávez-Béjar et al., 2013
*trc*	IPTG 0.1 mM	*MutmelA*		*Rhizobium etli*							Chávez-Béjar et al., 2013
*lac*	IPTG 0.1 mM	*aroG^*fbr*^*	pTrc99A	*Escherichia coli*	*Escherichia coli*	None	Glycerol	30°C	16.8	1.21	Mejía-Caballero et al., 2016
*PtktA*	None	*tktA*		*Escherichia coli*							Mejía-Caballero et al., 2016
*trc*	IPTG 0.1 mM	*antABC*		*Pseudomonas aeruginosa PAO1*							Mejía-Caballero et al., 2016
*trc*	IPTG 0.1 mM	*MutmelA*		*Rhizobium etli*							Mejía-Caballero et al., 2016

In another early example, the *Bacillus thuringiensis* strain 4D11 was shown to produce melanin when cultured for several hours with L-tyrosine at 42°C (Ruan et al., 2004). These results indicated that this organism should contain a gene encoding a tyrosinase in its genome. Since the sequence of the genome of *B. thuringiensis* 4D11 was not known, a cloning strategy was devised based on expected sequence similarity with a tyrosinase gene from *Bacillus cereus* 10987. A pair of PCR primers were designed based on the tyrosinase gene sequence from *B. cereus* 10987 and employed to amplify an 1,179 bp DNA fragment from *B. thuringiensis* 4D11 purified DNA. Sequence analysis showed this DNA fragment displayed 99% amino acid sequence similarity with the tyrosinase from *B. cereus* 10987. The PCR product was cloned in plasmid pGEM-7zf under the control of the *lac* promoter. Strain *E. coli* DH5α was transformed with this plasmid and the recombinant strain was shown to produce eumelanin at a titer of 5.6 g/L when grown in casein liquid medium (Table 1). Interestingly, it was also determined that this recombinant strain displayed a significantly higher survival rate when compared to DH5α, in experiments of exposure to UV-radiation (Ruan et al., 2005). These results show how in addition to conferring the capacity of producing melanin as a biotechnological product, the heterologous expression of a gene encoding a tyrosinase can increase the host's capacity to resist UV-radiation. This is the consequence of melanin production, a trait that can be beneficial in the case of microorganisms that are employed in the field, such as *B. thuringiensis*. The possibility of engineering microbes to survive in high-UV environments is also relevant for future space applications. Microbes are considered essential for helping to support human life by providing food, useful chemicals and recycling waste in long-range space missions and planet-colonization projects (Horneck et al., 2010; https://blogs.scientificamerican.com/observations/microbes-might-be-key-to-a-mars-mission/). Melanin can also absorb X and γ-rays, a characteristic that could increase the survival of engineered microbes in environments outside our planet.

Among soil bacteria, *Rhizobium etli* is especially important for agriculture since it can fix nitrogen when it forms nodules in the root of the plant *Phaseolus vulgaris*. It has been determined that this bacterium can produce melanin in the symbiotic nodules and a gene encoding a tyrosinase has been identified in a symbiotic plasmid (*melA*) (González et al., 2003; Piñero et al., 2007). The gene *melA* was cloned in the expression vector pTrc99A under control of the strong *trc* promoter and the resulting plasmid pTrc*melA* was transformed in *E. coli*. The recombinant *E. coli* strain produced eumelanin when L-tyrosine was provided as a substrate at 30°C and at a much lower quantity at 37°C (Cabrera-Valladares et al., 2006). It was also noted that melanin synthesis occurred only during the stationary culture phase. During cloning of the *melA* gene, a colony of recombinant *E. coli* in medium containing L-tyrosine was found to display a darker color when compared to the rest of the colonies. After DNA sequencing of the *melA* gene in this clone, it was determined that it had a spontaneous mutation of a single nucleotide change where the Asp535 residue was changed to a Gly residue in the MelA tyrosinase enzyme. This mutant version of MelA was named MutMelA. Further characterization revealed that eumelanin production in liquid cultures starts earlier in cultures of *E. coli* expressing MutmelA when compared to a strain expressing the wild type version of this enzyme. To develop and optimize a process for eumelanin production, a study was conducted to determine optimal condition for pigment synthesis in liquid cultures with a recombinant *E. coli* strain expressing MutmelA. The effect of the concentration of antibiotic for plasmid selection pressure, isopropyl-d-thio-galactopyranoside (IPTG) as gene inducer, culture temperature and pH on eumelanin concentration were determined. The best conditions for production in bioreactor consisted on the use of 0.1 mmol/L of IPTG, a culture temperature of 30°C and changing the pH of the medium from 7.0 to 7.5 at the start of the eumelanin production phase. A total of 6 g/L of L-tyrosine was added to the culture medium as eumelanin precursor. Under these conditions, a 100% conversion yield of L-tyrosine to eumelanin was observed with a final titer of 6 g/L (Table 1) (Lagunas-Muñoz et al., 2006). These results highlight the importance of culture conditions optimization as a factor for reaching the maximum yield and productivity with a recombinant melanogenic strain.

In a bioprospecting study, microorganisms with the capacity of producing melanin were isolated from soil samples in China. One of such microbes was identified as *Streptomyces kathirae* SC-1, it displayed the highest capacity for melanin production among all isolates. A surface response method was employed to optimize medium and growth conditions, allowing the production of 13.7 g/L of melanin (Guo et al., 2014). It is important to point out that the culture medium employed in this study included yeast extract, which provided a mixture of melanin precursors. Therefore, the resulting polymer should be characterized to determine its chemical composition to define the type of melanin produced. To better understand melanogenesis in this organism, a novel tyrosinase was purified to homogeneity. This is a 30-kDa enzyme, displaying K_m_ for L-DOPA and L-tyrosine of 0.42 and 0.25 mM, respectively. The partial amino acid sequence of this tyrosinase was employed to design primers that allowed the amplification of the encoding *melC* gene and its promoter region. Sequence analysis of the promoter region identified two putative promoters: P_skmel_ and P_135_. The gene *melC* was cloned under the transcriptional control of either putative promoter and the constitutive promoter *Perm*E^*^ in the replicative plasmid pIJ86 and the resulting constructs transformed in *S. lividans* and *S. kathirae*. The recombinant strains of *S. lividans* were characterized, and it was determined that P_skmel_ is the functional promoter for *melC*. The recombinant strains of *S. kathirae* were cultured under melanin production conditions. It was determined that strains expressing *melC* from *Perm*E^*^ or P_skmel_ produced 24.9 and 28.8 g/L of melanin, respectively (Table 1) (Guo et al., 2015). It should be noted that these are the highest melanin titers reported to date, highlighting the potential of applying genetic engineering techniques to further enhance the production capacity of a melanogenic organism (Table 1). This production system has the potential for further optimization, in particular regarding culture medium composition. The medium contains a relatively high amount of yeast extract (37 g/L), which is a costly component. Yeast extract could complicate melanin purification procedures and some of its components can react with melanin precursors, yielding a polymer not composed entirely of the L-tyrosine precursor. For these reasons, the search for a culture medium containing only salts and a simple carbon source should be a future research objective to improve the current production scheme.

**Figure 3:**
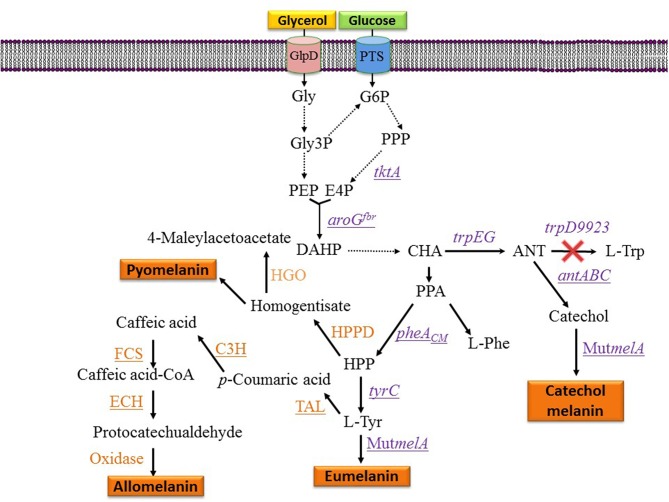
Metabolic pathways and expressed genes related to the synthesis of melanins with engineered microorganisms. Dashed arrows indicate two or more enzyme reactions. Underlined genes were overexpressed from plasmids. PTS, phosphotransferase system glucose transport protein; Gly, glycerol; Gly3P, glycerol-3-phosphate; G6P, glucose-6-phosphate; E4P, D-erythrose 4-phosphate; PEP, phosphoenolpyruvate; DAHP, 3-deoxy-D-*arabino*-heptulosonate 7-phosphate; HPP, 4-hydroxyphenylpyruvate; CHA, chorismate; ANT, anthranilate; PPA, phenylpyruvate; HPPD, hydroxyphenylpyruvate dehydrogenase; HGO, homogentisate 1,2-dioxygenase; L-Tyr, L-tyrosine; L-Phe, L-phenylalanine; L-Trp, L-tryptophan; *tktA*, gene encoding transketolase; *aroG*^*fbr*^, gene encoding feedback inhibition resistant DAHP synthase; *trpEG*, genes encoding anthranilate synthase component I; *trpD9923* is a mutant version of *trpD* causing the loss of anthranilate phosphoribosyl transferase activity and retaining anthranilate synthase activity; *tyrC*, gene encoding cyclohexadienyl dehydrogenase; C3H, gene encoding *p*-coumarate 3-hydroxylase; TAL, gene encoding tyrosine ammonia-lyase; FCS, gene encoding feruloyl-CoA synthetase form *B. glumae* BGR1; ECH, gene encoding enoyl-CoA hydratase/aldolase from *B. glumae* BGR1; *antABC*, encodes the terminal oxygenase and the reductase components of anthranilate 1,2-dioxygenase from *P. aeruginosa* PAO1; *pheA*_*CM*_, gene encoding chorismate mutase domain from chorismate mutase-prephenate dehydratase; Mut*melA*, gene encoding a mutant version of the tyrosinase from *R. etli*.

The phenolic aldehydes are compounds having applications in the chemical and food industries. The microbial production of this class of chemicals in *E. coli* involves the expression of heterologous genes and other modifications to the metabolic network. As part of a study to generate an *E. coli* strain for the synthesis of phenolic aldehydes, this organism was modified to produce caffeic acid from L-tyrosine. This involves the expression of tyrosine ammonia-lyase (TAL) to transform L-tyrosine to coumaric acid and *p*-coumarate 3-hydroxylase (C3H) to produce caffeic acid (Figure 3). In these experiments, a dark pigment was observed, having the characteristics of melanin. This caffeic acid melanin is likely produced by oxidation of the catechol moiety by some of the oxidases encoded in the genome of *E. coli*. It was also observed that protocatechualdehyde added to the culture medium and incubated with *E. coli* yielded a melanin pigment with a brown color, whereas caffeic acid melanin was black. As part of this work, the genes encoding feruloyl-CoA synthetase (FCS) and enoyl-CoA hydratase/aldolase (ECH) from *Burkholderia glumae* BGR1 were expressed in *E. coli* (Figure 3). The recombinant strain acquired the capacity to convert caffeic acid to protocatechualdehyde. As part of this study, it was observed that in the presence of 5 mM caffeic acid, wild type *E. coli* BL21(DE3) produced 0.15 g/L of melanin (Table 1). When the same amount of caffeic acid was added to a culture with a recombinant strain expressing *fcs* and *ech*, melanin was produced at a faster rate, reaching a titer of 0.2 g/L (Jang et al., 2018). This melanin product was not chemically characterized, it is likely a polymer composed of a mixture of caffeic acid and protocatechualdehyde moieties. These results demonstrate the production of caffeic acid and protocatechualdehyde melanins with recombinant *E. coli*. It evident that FCS and ECH activities have an influence on the synthesis of melanin and/or melanin precursors in this strain, however, the mechanisms for the observed results are not yet completely understood. The chemical characterization of the produced melanin should provide further insight into the chemical precursors involved in its formation. It should also be of interest to identify the native enzyme from *E. coli* that is involved in the oxidation of caffeic acid and protocatechualdehyde, leading to their polymerization into melanin. The cloning and overexpression of the gene encoding this yet unidentified oxidase should enable improvement of melanin-producing strains. In a subsequent report, it was demonstrated that the protocatechualdehyde-based melanin could be employed to dye soft contact lenses (Ahn et al., 2019). The antibacterial and antioxidant activity of melanins should be advantageous in such an application when compared to chemically synthesized dyes.

## Random Mutagenesis for the Selection of a Melanogenic Strain

Strain F6 of the soil bacterium *Pseudomonas putida* was found to display the capacity of producing melanin when grown in media containing L-tyrosine. To gain insight into the role of genes involved in melanogenesis, transposon mutagenesis was performed. This process yielded two mutants with increased melanin production capacity. One of such mutants (F6-HDO) produced 0.35 g/L of melanin, which corresponds to a 6-fold increase when compared to *P. putida* F6 (Table 1). Interestingly, this mutant displayed higher resistance to UV light and H_2_O_2_ when compared to the wild type. Genetic analysis indicated that transposon mutagenesis disrupted a gene encoding HGA 1,2-dioxygenase (HGO). This enzyme converts HGA into 4-maleylacetoacetate as part of a degradation pathway. Therefore, this mutation is expected to reduce HGA consumption by HGO. This result indicates that HGA is the allomelanin precursor in this mutant strain (Figure 3) (Nikodinovic-Runic et al., 2009). The synthesis of HGA originates from the L-tyrosine biosynthetic pathway. The intermediate 4-hydroxyphenylpyruvate (HPP) is transformed into HGA by enzyme hydroxyphenylpyruvate dehydrogenase (HPPD) (Figure 3). This is an example where random mutagenesis was employed to isolate mutants with improved melanogenesis. An important advantage of working with melanogenic organisms is the simplicity in the process for identifying mutants since they can be detected visually. Studies, such as this one are essential for identifying novel genes involved in the melanogenesis process. Once the melanogenic pathways are identified, a rational strategy can be applied to enhance the native melanogenic capacity or transfer it to another organism.

Random mutagenesis is a relatively simple method for strain improvement but it is limited to organisms that already have a native melanin production capacity. Usually, the site and type of mutation in the improved melanogenic organism are not known, thus limiting the use of rational strategies for further strain improvement. In addition, the genetic changes produced by random mutagenenis can be unstable so the strain could revert to a low producer phenotype. A solution to these issues can be based on genome sequencing of the improved strain, yielding information about the type of mutation as well as the genes and pathways involved in the observed phenotype. This information can be employed to “reverse-engineer” the melanogenic organism by employing genetic engineering techniques to reintroduce the identified mutations. This strategy can be employed to separate the genetic changes that are related to the improved phenotype from those that could be deleterious or resulting from genetic instability.

The previous examples described recombinant strains and processes for the conversion of diverse aromatic compounds into melanins. By adding various melanin precursors into the culture medium, they can be employed by tyrosinases as substrates to generate specific pigments (Table 1). Such processes have the potential for displaying high productivity. Furthermore, by employing diverse aromatic precursors, various types of melanins can be produced. In spite of these advantages, a few drawbacks can be considered. One of them is the relatively high cost of employing pure melanin precursors. However, another problem is created when non-pure and relatively inexpensive melanin precursors are employed, such as yeast extract or protein hydrolysates. The use of complex media can result in variability in the composition of produced melanins, since these culture media can contain diverse and variable amounts of compounds that can be substrates of tyrosinases or that can react with melanin precursor molecules. Furthermore, the use of non-defined media makes melanin purification processes more difficult and expensive.

The genetic modifications employed to generate the previously described production strains are mostly based on the cloning of the genes encoding a tyrosinase on a multicopy expression plasmid (Table 1). This approach proved to be effective for achieving titers in the scale of grams in several examples. However, it remains to be determined if the chromosomal expression of these genes could lead to efficient production strains, having the advantage of not requiring the use of antibiotics for plasmid selection. It should be noted that *E. coli* has been chosen frequently as a production host for melanins. This is likely a result of the extensive set of genetic and metabolic engineering tools available for this organism (Table 1). However, potential advantages of engineering natural melanogenic organisms should be taken into consideration. The highest melanin titer reported to date was generated in a process with a recombinant strain of *S. kathirae*. It could be expected that melanogenic organisms have physiological traits that make them more suitable as production strains. For example, specialized metabolic pathways for the generation of melanin precursors, enhanced transport processes for the internalization of tyrosinase substrates or for the excretion of melanin.

## Metabolic Engineering Applied for the Production of Melanins From Simple Carbon Sources by Increasing Precursor Supply

One potential solution to the issues mentioned above involves the generation of microbial strains for the total synthesis of melanins from simple carbon sources. This approach is based on applying metabolic engineering strategies to increase flux into the shikimate pathway which provides the precursors for the aromatic amino acids. In one example, metabolic engineering methods were applied to generate an *E. coli* strain with the capacity of producing the eumelanin precursor L-tyrosine from glucose (Chávez-Béjar et al., 2008). This strain was modified to increase carbon flow to the L-tyrosine biosynthetic pathway by overexpressing the genes encoding a feedback-insensitive version of the enzyme 3-deoxy-D-*arabino*-heptulosonate 7-phosphate (DAHP) synthase (*aroG*^*fbr*^), cyclohexadienyl dehydrogenase (TyrC) from *Zymomonas mobilis* and the chorismate mutase domain from the native enzyme chorismate mutase-prephenate dehydratase. In addition, this strain expressed the gene Mut*melA* encoding the tyrosinase MutMelA (Figure 3). This strain had the potential for synthesizing eumelanin from glucose. However, it was determined that MutMelA activity depleted the L-tyrosine pool, causing a defect in cell growth. The enzyme tyrosinase requires Cu as a cofactor for activity. Therefore, this element was left out of the medium during the first half of the culture to avoid L-tyrosine depletion by MutMelA. The eumelanin production phase was started by adding CuSO_4_ to the medium, causing the activation of tyrosinase. This strategy was employed in bioreactor cultures with medium containing 60 g/L of glucose as the sole carbon source. In 120 h, 3.2 g/L of eumelanin were produced (Table 1) (Chávez-Béjar et al., 2013). These results were the first example where metabolic engineering was applied to generate a strain for the total synthesis of eumelanin. This study provided useful information regarding the potential negative consequences in cell physiology resulting from the high-level expression of tyrosinase. This problem was alleviated by adopting a delayed activation of the heterologous enzyme. An alternate solution might be based on fine control of gene induction at a specific phase in the production culture.

During the characterization of enzyme MutMelA it was determined that in addition to L-tyrosine, it can also employ catechol as a substrate. Thus, this enzyme could be employed for the synthesis of catechol melanin. To test this idea, a bioconversion process was developed with an *E. coli* strain expressing MutMelA and growing in medium containing glycerol 40 g/L as the carbon source and catechol 0.85 g/L as tyrosinase substrate. After 54 h, 0.29 g/L of catechol melanin were produced. To further improve this process, metabolic engineering was evaluated to generate a strain with the capacity of generating catechol melanin from a simple carbon source. The strategy that was followed is based on employing an engineered *E. coli* strain that can produce catechol from a simple carbon source (Balderas-Hernández et al., 2014). Strain *E. coli* W3110 *trpD9923* is a mutant in the L-tryptophan biosynthetic pathway that overproduces the intermediate anthranilate (Yanofsky et al., 1971). This strain was modified to increase carbon flow to anthranilate by overexpressing genes *aroG*^*fbr*^ and *tktA*, encoding a feedback-insensitive version of DAHP synthase and transketolase, respectively (Figure 3). These modifications caused a 2-fold increase in anthranilate titer in flask cultures (Balderas-Hernández et al., 2009). This strain was further modified by the expression of the genes *antABC* encoding anthranilate 1,2-dioxygenase from *Pseudomonas aeruginosa* PAO1. This enzyme catalyzes the conversion of anthranilate to catechol (Figure 3). In the final step of strain construction, the gene Mut*melA* was integrated into the chromosome at the site of the *lacZ* gene. The resulting strain was evaluated in bioreactor cultures at 1-liter scale. The culture media contained glycerol 40 g/L as the carbon source. Glycerol was chosen over glucose as the carbon source since the former does not consume aromatics precursor PEP during its internalization and phosphorylation. In addition, glycerol is a relatively inexpensive, abundant and renewable carbon and energy source that is obtained mainly as a byproduct of biodiesel and soap production (Tan et al., 2013). Culture media also contained 2 g/L yeast extract since the strain is an L-tryptophan auxotroph. Under these conditions, the engineered strain displayed growth for 17 h then it entered the stationary phase that ended after 72 h of total culture time. The accumulation of catechol melanin was observed to begin at 18 h, very close to the start of the stationary phase. At the end of the culture, 1.21 g/L of catechol melanin were recuperated from the culture medium (Table 1) (Mejía-Caballero et al., 2016). The accumulation of 0.73 g/L of catechol was observed at the end of the culture. This result indicates that the rate of synthesis of this precursor surpasses the capacity of MutMelA to consume it. Therefore, in this case, increasing the activity of the tyrosinase should be a target to improve strain performance.

Metabolic engineering efforts to increase melanin production have so far focused on *E. coli*. This is the result of the accumulated knowledge related to the engineering of central metabolism and the shikimate pathway in this organism. For the yeast *Saccharomyces cerevisiae*, there is also a large body of work related to the rational modification of metabolic pathways for the production of aromatic compounds. Some of these modifications have been directed to increase the supply of L-DOPA since this compound is an early intermediate for the synthesis of benzylisoquinoline alkaloids (BIAs). In one report, with the aim of improving an *S. cerevisiae* strain for the production of BIAs, a strategy based on the use of an enzyme-coupled biosensor and mutagenesis was employed. The cytochrome P450 L-DOPA oxidase CYP76AD1 from the sugar beet *Beta vulgaris* was found to display tyrosine hydroxylase activity, leading to the synthesis of L-DOPA. To improve this activity, error-prone PCR was employed to generate a mutant library of CYP76AD1. The identification of mutants with higher activity was based on the visual detection of colonies displaying the highest fluorescence since the cells express an enzyme that converts L-DOPA to betaxanthin. In a second step, DNA shuffling was employed with the genes of the six isolated improved variants of CYP76AD1 to combine the mutations. This procedure allowed the isolation of a mutant version of CYP76AD1 that displayed a 2.8-fold increase in L-DOPA titer when compared to wild type enzyme (DeLoache et al., 2015). In another example, *S. cerevisiae* strains were engineered for the synthesis of natural and novel BIAs. The simultaneous deletion of *zwf1*, encoding glucose-6-phosphate dehydrogenase, upregulation of *TKL1*, encoding transketolase and the expression of *ARO4*^*Q*166*K*^, encoding a feedback-inhibition-resistant mutant version of the tyrosine-inhibited DAHP synthase, improved the endogenous supply of L-tyrosine, leading to a 60-fold increase in the synthesis of the benzylisoquinoline scaffold. In an effort to generate a strain for the production of norcoclaurine, further modifications were introduced to enable the synthesis of L-DOPA. The BH_4_-dependent tyrosine hydroxylase from *Rattus norvegicus* was chosen. Codon-optimized genes encoding enzymes involved in BH_4_ biosynthesis and tyrosine-hydroxylase were expressed, resulting in the synthesis of 94.5 ug/L of L-DOPA.

It should be noted that these efforts were not aimed exclusively at generating *S. cerevisiae* strains for L-tyrosine or L-DOPA production. Thus, further performance improvement should be possible. It is expected that expression of an enzyme with tyrosinase activity in these strains should yield eumelanin producers. It is interesting to note the similarities and differences regarding metabolic engineering targets when comparing *E. coli* and *S. cerevisiae* L-tyrosine or L-DOPA-production strains. One clear similarity is the need to express feedback-inhibition-resistant mutant versions of enzymes in key points of the aromatic biosynthetic pathways.

## Conclusions and Perspectives

The melanins are a class of natural products that can be considered functional polymers with multiple potential applications in industry. Obtaining these products at a large scale, with a chemically defined composition, and at a relatively low cost is a major technical challenge. As discussed in this review, one approach in this direction can be based on the isolation and use of natural melanogenic microorganisms. This scheme has some advantages, such as the possibility of developing a production process in a relatively short time. However, the use of natural melanogenic organisms can have some drawbacks, such as the frequent requirement to use complex media, which is required to induce melanin production. The use of complex media complicates purification procedures and also can result in the synthesis of melanin with non-desired chemical components. One solution to these problems has been based on the use of genetic engineering to modify the expression of native genes involved in melanogenesis, as well as the generation of novel melanogenic organisms. The accumulated knowledge on the biochemistry and genetics of melanin production in various organisms has enabled the possibility of directly manipulating components in this pathway. By employing genetic and metabolic engineering techniques, it has become possible to enhance the synthetic capacity of natural melanogenic organisms. Furthermore, novel melanogenic organisms have been generated with the capacity of synthesizing melanins from simple carbon sources. These efforts have resulted in the generation of strains and processes for obtaining these polymers at the scale of grams (Table 1).

The main genetic modification employed to generate or improve melanogenic organisms involves the overexpression of genes encoding tyrosinases. This is frequently based on placing the tyrosinase gene under control of an inducible promoter in a replicative plasmid vector. This strategy enables the precise control of the magnitude and time of gene expression by the addition of inducers, thus allowing production process optimization. However, the use of expression plasmids like those employed in the examples reviewed here requires the addition of antibiotics as a selective pressure to avoid the growth of plasmid-less cells. Another drawback is the requirement for the inclusion of a chemical inducer in culture media. The use of antibiotics and inducers increase production costs and complicates purification procedures. These issues can be avoided by the use of alternative plasmid selection methods that are not based on antibiotics, as well as gene induction methods not dependent on the addition of chemicals (Vidal et al., 2008).

It can be observed in several of the reports reviewed here, that melanin titers and volumetric productivities are lower in processes where the production strain was modified by metabolic engineering to convert the carbon sources to melanins when compared to the strains that transform melanin precursors provided in the culture medium (Table 1). The reported titers and productivities for eumelanin fall short of those observed for the production of its precursor L-tyrosine (Santos et al., 2012). This suggests that there is still a potential margin for strain and production process improvement. Further development of the engineered strains will be required to make them more competitive.

The application of synthetic biology, adaptive laboratory evolution (ALE) and mutagenesis strategies should be evaluated for improving the current melanin production strains (Bassalo et al., 2016). The use of ALE can allow the engineering of complex phenotypes. In one report, a synthetic biosensor module that responds to aromatic amino acids intracellular concentration was combined with ALE to allow the generation of an improved *S. cerevisiae* strain for muconic acid production (Leavitt et al., 2017). This strain displays enhanced flux in the common aromatic amino acid pathway, thus, it could be modified to increase L-tyrosine synthesis by following established methods. With such modification, the *S. cerevisiae* strain developed in this study could be a suitable platform for eumelanin synthesis. In another report, a high-throughput screen for L-tyrosine production was developed by coupling the synthesis of this amino acid to the production of melanin in an *E. coli* strain expressing the MelA tyrosinase from *R. etli* (Santos and Stephanopoulos, 2008). This method was applied to identify *E. coli* strains with improved L-tyrosine production capacity. In this study, *E. coli* was engineered by applying rational metabolic engineering strategies that cause L-tyrosine overproduction. To further improve L-tyrosine synthesis capacity, this strain was subjected to global transcription machinery engineering (gTME) (Alper et al., 2006). This method was implemented in *E. coli* by expressing in the engineered strain two separate gTME libraries of the RNA polymerase *rpoA* and *rpoD* subunits. Improved L-tyrosine producers from these two libraries were identified in agar plates based on colony melanin pigmentation. Three mutant isolates were characterized, showing a 2-fold increase in L-tyrosine titer when compared to the engineered parent strain (Santos et al., 2012). It should be noted that in this case, these strains could be employed directly in a process for melanin production from glucose.

As part of the characterization of strains modified to synthesize melanin from a simple carbon source, it has been determined that tyrosinase activity is a factor limiting productivity (Chávez-Béjar et al., 2013; Mejía-Caballero et al., 2016). It is possible that tyrosinase activity could also be limiting melanin production in other engineered strains. It is, therefore, of importance, to evaluate tyrosinase enzymes from diverse biological sources, to identify those with desired properties for biotechnological application. The vast genome and metagenome data that is currently available should provide a large number of genes encoding putative tyrosinases that can be evaluated experimentally. In addition, the application of protein engineering is a viable option to improve this class of enzymes. This methodology has not yet been applied as part of a strategy to improve a melanin production strain. One important advantage of working with tyrosinases is the simple activity assay based on melanin production, which allows high-throughput selection methods (Santos and Stephanopoulos, 2008).

In spite of the technical advances regarding the development of strains and processes for melanin production, many basic questions still remain to be answered. One important issue is related to the dynamics of melanin polymerization. It is assumed that melanin precursors are synthesized in the cytosol, these molecules then exit the cell and start to polymerize in the culture medium. The polymer progressively increases in size, generating a large diversity of melanin molecules. It is interesting that melanin isolated at different times in production cultures, display diverse colors ranging from yellow to black (Chávez-Béjar et al., 2013). It can be expected that these macromolecules will also have distinct physicochemical properties. Performing studies on the dynamics of melanin polymerization in production cultures and the properties of polymers of particular sizes is of great importance since they could yield useful information leading to the isolation of products with defined characteristics.

To be used as a biotechnological product, melanins must be extracted from culture media and purified. A general method for extracting and partially purifying these products is based on the low solubility displayed by these polymers at low pH values. The extraction method followed by most authors starts by removing cells from the culture medium by centrifugation and then precipitation of melanin by adjusting pH to 2.0–3.0 with HCl for 4–16 h at 4–25°C. Precipitated melanin is centrifuged and it can be either dried in an oven at 45–70°C for 24 h or freeze-dried and stored at 4°C. Alternatively, the precipitated melanin can be re-dissolved in water at pH 8.0–9.0 and the cycle of precipitation and re-dissolving is repeated several times with drying as a final step. Liquid chromatography by Pharmacia Sephadex LH-20 has been reported as an additional purification step for eumelanin (Lin et al., 2005). These extraction and purification methods are expected to yield melanins with varying degrees of purity. It is likely that melanin obtained with the previously mentioned procedures could contain varying amounts of protein and other cellular components. However, there is still not a general standard to define melanin purity for specific applications.

As it is evident from the manuscripts reviewed here, most of the published works on microbial melanin production have focused on eumelanin. This is understandable since this polymer has been characterized extensively and it is the most common type of melanin found in humans. Therefore, eumelanin availability could lead to applications in the cosmetic and health industries as well as other technological areas. However, it should be noted that melanins comprise a chemically-diverse group of polymers. So far, only a small fraction of this chemical diversity has been explored. In addition to eumelanin, production processes for catechol, caffeic acid, and protocatechualdehyde melanins have been reported. For specific applications, it can be assumed that different types of melanins would display distinct performances. Indeed, in a recent study, it was shown that protocatechualdehyde-based melanin displayed a better performance as a dye in soft contact lenses, when compared to eumelanin or caffeic acid melanin (Ahn et al., 2019). It should also be noted that non-natural melanins can be generated by employing synthetic non-natural amino acids and other compounds that can be employed as substrates by tyrosinases (della-Cioppa et al., 1990). Therefore, the expected diversity of this type of polymers is very large. The development of strains and processes for generating novel natural and synthetic melanins should vastly increase the number of applications with these aromatic polymers.

## Author Contributions

LM, AM, and GG participated in the search and analysis of information for this review as well as in writing and critical review of the manuscript.

### Conflict of Interest

The authors declare that the research was conducted in the absence of any commercial or financial relationships that could be construed as a potential conflict of interest.
